# A deep learning model to triage and predict adenocarcinoma on pancreas cytology whole slide imaging

**DOI:** 10.1038/s41598-023-42045-w

**Published:** 2023-10-02

**Authors:** Andrew Sohn, Daniel Miller, Efrain Ribeiro, Nakul Shankar, Syed Ali, Ralph Hruban, Alexander Baras

**Affiliations:** 1 Department of Pathology, Johns Hopkins University School of Medicine, Baltimore, MD USA; 2 Department of Pathology, Saint Louis University School of Medicine, St. Louis, USA; 3 Department of Pathology, University of Colorado, Boulder, USA

**Keywords:** Cancer imaging, Machine learning

## Abstract

Pancreatic fine-needle aspirations are the gold-standard diagnostic procedure for the evaluation of pancreatic ductal adenocarcinoma. A suspicion for malignancy can escalate towards chemotherapy followed by a major surgery and therefore is a high-stakes task for the pathologist. In this paper, we propose a deep learning framework, MIPCL, that can serve as a helpful screening tool, predicting the presence or absence of cancer. We also reproduce two deep learning models that have found success in surgical pathology for our cytopathology study. Our MIPCL significantly improves over both models across all evaluated metrics (F1-Score: 87.97% vs 88.70% vs 91.07%; AUROC: 0.9159 vs. 0.9051 vs 0.9435). Additionally, our model is able to recover the most contributing regions on the slide for the final prediction. We also present a dataset curation strategy that increases the number of training examples from an existing dataset, thereby reducing the resource burden tied to collecting and scanning additional cases.

## Introduction

Over the past few years, there has been an explosion of studies that have shown the impressive and exciting utility of deep neural networks for the classification of digital whole slide images (WSIs) of surgical pathology specimens^1–7^. However, comparatively little has been done for cytopathology WSIs^8–12^. There are many important differences between the two that have defining consequences for building appropriate deep learning models. Broadly speaking, surgical pathology involves the examination of two-dimensional slices of tissue sampled from intact tissue, biopsies or resections. Cytopathology, on the other hand, involves the examination of single cells or a three-dimensional (via a Z-plane) group of cells from a suspected pathological lesion. Cytopathology is further partitioned based on the technique for specimen collection (lavage, brushing, smear), and on the specimen preparations for expert review.

Obtaining a cytopathology specimen is typically less invasive (i.e., can be obtained in a clinic or physician’s office) than that of a surgical pathology specimen, and cytopathology is therefore more amenable for regular screening and early diagnosis. While cytopathological testing is more readily available, it comes at the expense of important features that can be crucial, for example, towards subtyping cancer and identification of molecular characteristics that can inform management. Therefore, cytopathology excels in scenarios where the diagnostic specifics are not as important at that point in time. Rather, the immediate goal is to initiate a management plan, which will differ significantly depending on whether the disease at hand is neoplastic or not.

Tumors of the pancreas are extremely difficult to diagnose because the organ sits deep in the abdomen and is shrouded by the other surrounding organs. Although various radiographic studies may reveal a mass in the pancreas, the most accurate way to diagnose pancreatic cancer is by examining an endoscopic ultrasound-guided biopsy microscopically^13,14^. These biopsies can be sampled via two modalities, a fine needle aspiration (FNA) or a fine needle biopsy (FNB). While FNBs with the latest needle technology have the potential to become the preferred modality in the future, FNAs are still widely utilized over FNBs. The presence of cancer on the biopsy may cascade to a Whipple procedure—an operation to remove the head of the pancreas, most of the duodenum, a portion of the bile duct, the gallbladder, and associated lymph nodes—thereby making the correct diagnosis a high-stakes endeavor for the pathologist.

To date, only a small handful of studies have shown applicability of deep learning for pancreatic FNAs^12,15,16^. Zhang et al.^12^ utilized a more conventional deep convolutional neural network (DCNN) for the tasks of both segmenting and classifying tumor on the slide. The authors report that their DCNN achieved an area under the receiver operating characterstic curve (AUROCs) of 0.958 and 0.948–0.976 on internal and external testing, respectively, for the classification of pancreatic cancer. Zhang et al.^15^ and Zhang et al.^16^ both use more recent vision transformer based neural networks, and report F1-scores of 93.94% and 91.32%, respectively, for the classification of pancreatic cancer. All three studies trained and evaluated on internally curated datasets, and therefore, it is difficult to compare one another.

Most importantly, however, all three studies did not investigate at the level of WSI, and only examined Diff-Quik stains (pancreas FNAs typically come with both Diff-Quik and Pap smears). The datasets from these three studies were constructed from digital snapshots of subregions on the slide, at 40$\times$ resolution, which included adenocarcinoma cells or normal cells. These images were were acquired by digital cameras mounted on microscopes by senior cytopathologists while reviewing the slides from pancreatic FNA cases. The snapshot images from these studies ranged from 1390 $\times$ 1038 pixels to 5480 $\times$ 3648 pixels.

While all three studies proposed new and respectable deep learning models (convolution based or vision transformer based) for their respective classification tasks, they did not have to devise methodologies to deal with ultra-high resolution, which is the scale where digitally scanned cytopathology slides reside. A digitally scanned cytopathology slide would be on the order of approximately 50,000–100,000 pixels per X and Y dimensions. This high gigapixel resolution is arguably the major pain point of developing deep learning models for digital pathology (surgical- and/or cytopathology). We conducted our study over digitally scanned regional whole-slide images (averaging 15,000 $\times$ 15,000 pixels) of pancreatic FNA cases, collected from our institution, to mimic real-world scenarios. Furthermore, our dataset consisted of both Diff-Quik and Pap smear preparations.
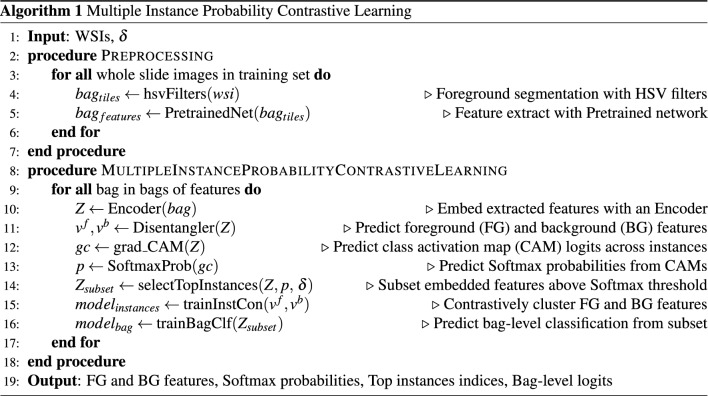


In this study we developed and tested a deep learning algorithm, multiple instance probability contrastive learning (MIPCL), for the classification of pancreatic ductal adenocarcnoma (PDAC) on WSIs of pancreas FNAs. Along the way, we reproduced two deep learning models that have found success in the domain of surgical pathology—attention-based multiple instance learning (ABMIL)^17^ and clustering-constrained attention multiple instance learning (CLAM)^6^—to serve as models of comparison to our proposed MIPCL model. While we are happy to report that both ABMIL and CLAM works well for digital cytopathology, MIPCL significantly improves over both ABMIL and CLAM across all evaluated metrics. All three models are able to perform model explainability and visualization.

## Results

### Cross-validated model performances

**Figure 1:**
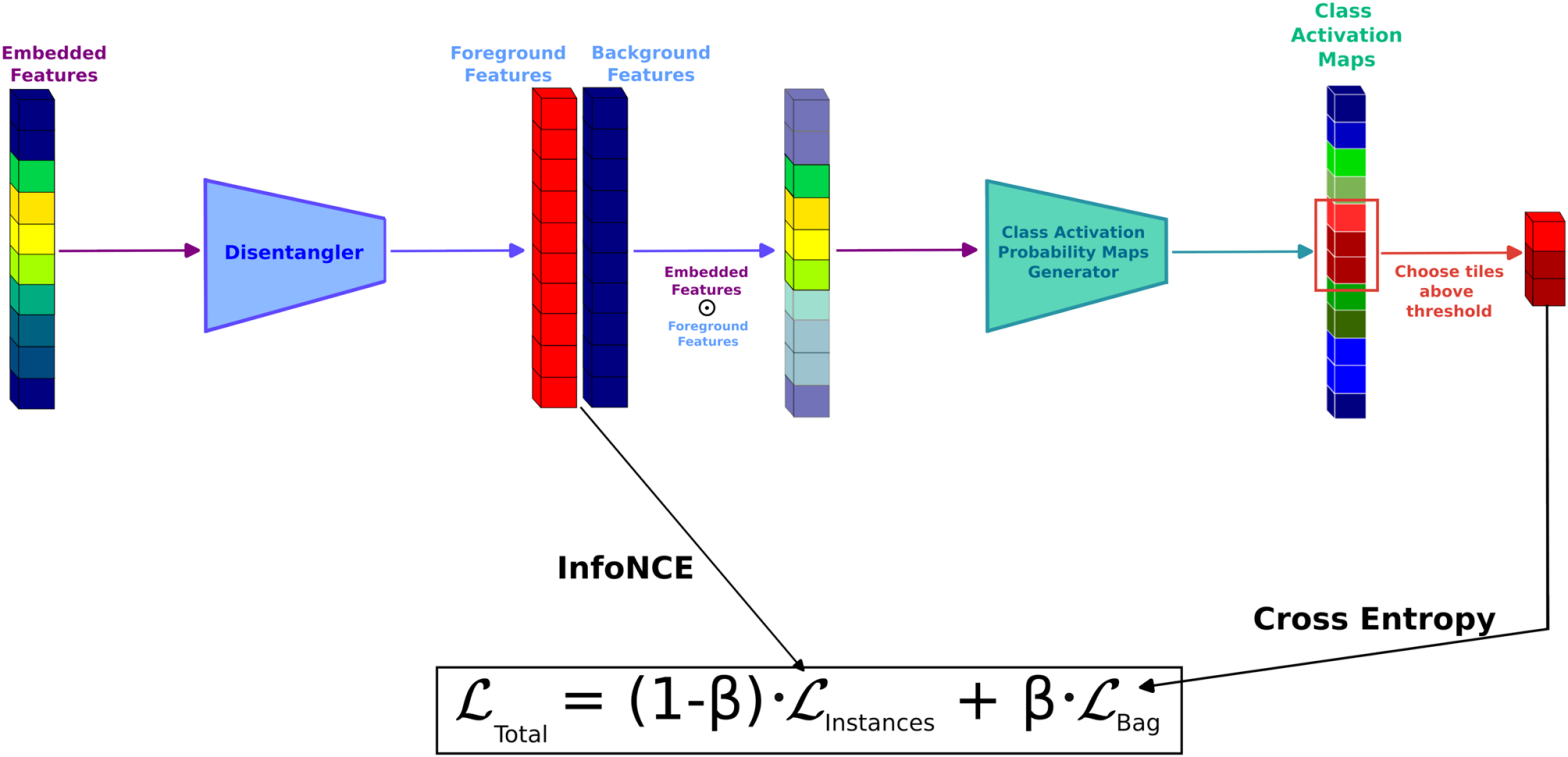
Overview of our MIPCL framework. The extracted features from the preprocessing step is first encoded with a simple MLP network and embedded into a lower dimension. The outputs of the encoder are then passed through a disentangler module, which predicts only one class-agnostic activation map per instance, and thereby, generating a foreground and a background for each instance. The foreground and background are contrasted from one another via the InfoNCE loss funtion. An Einstein summation is then performed between the original encoded features and the foreground, and a class activation map is generated from each instance. The class activation map is then converted to Softmax probabilities, and the instances above a threshold probability are selected out and pooled for a final bag-level classification with the Cross-Entropy loss function. A hyperparameter $\beta$ is used to weight the two losses for a total loss function.

We evaluated the slide-level classification performance of MIPCL for detecting the presence of pancreatic adenocarcinoma using a stratified 10-fold cross-validation. For each cross-validated fold, we randomly split our pancreatic FNA dataset into a training set (80%), a validation set (10%), and a test set (10%), stratified by the two class labels. Due to the imbalanced nature of our dataset (section “Data curation”), we also implemented a weighted batch sampler—with respect to the slide label ratios—for the dataloader during training. For each training fold, we monitor the total loss (Fig. 1) of the validation set for model selection. Inference on the test set is performed once, at the end of training, once a model has been selected. As for evaluation metrics, we looked at balanced accuracy, weighted F1-Score, and the weighted area under the curve (AUC) for both the receiver operating characteristic (AUROC) and the precision-recall (AUPRC). Statistical significance and confidence intervals were evaluated on the test sets between the models with Wilcoxon signed-rank test and bootstrapping, respectively.

While each of the WSI from our pancreatic cytopathology dataset come with anywhere from 10 to 13 Z-planes (some cases didn’t scan properly for all thirteen planes), we found that using the initial in-focus Z-plane (the zero plane) was sufficient for our classification task. Moreover, utilizing a single plane was vastly more economical with respect to computational costs (memory and speed); a single graphic processing unit (GPU) was more than sufficient to train a single z-plane versus requiring more than 4 GPUs to train all Z-levels. We also observed that the initial zero plane performed best among all the available Z-planes.

**Figure 2:**
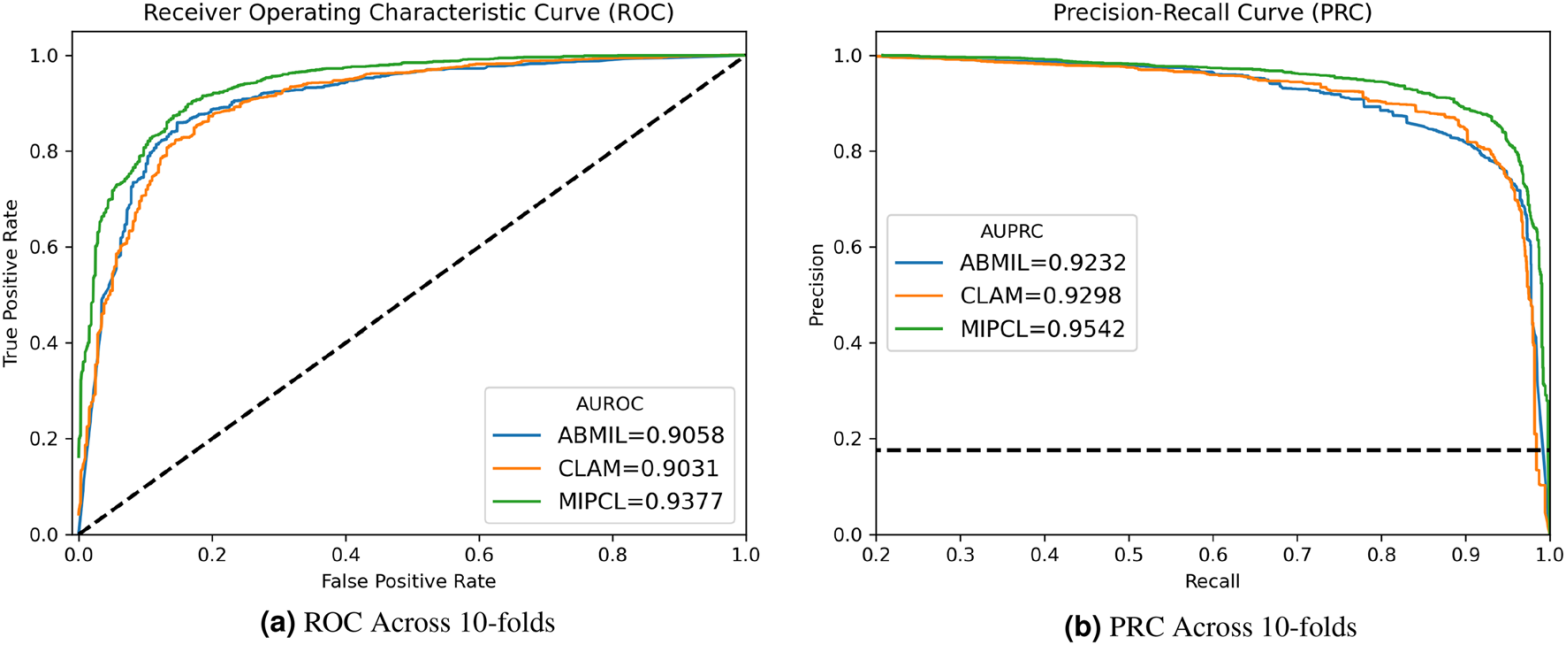
Receiver operating characteristic and precision-recall curves compared across the three models. (**a**) While all three models achieve high balanced prediction accuracies, our MIPCL model performed the best (0.9377). (**b**) All three models also achieve high performance with regards to how well true positives are identified, and again with our MIPCL model performing best (0.9542).

We report that both ABMIL and CLAM can be recapitulated for digital cytopathology classification, and importantly, both models perform well. Because CLAM is essentially an extension over ABMIL for digital surgical pathology, it was unsurprising to observe that CLAM also improved over ABMIL for the purposes of digital cytopathology as well. For our classification task, across 10 test folds, ABMIL had a balanced accuracy of 88.12%, a weighted F1-score of 87.98%, a weighted AUROC of 0.9058, and a weighted AUPRC of 0.9232. CLAM had following scores of 89.06% (+ 0.94), 89.05% (+ 1.07), 0.9031 (− 0.27), and 0.9298 (+ 0.66), respectively. Our MIPCL framework improves over both ABMIL and CLAM, across all metrics (Table 1), and by a significantly larger margin than that of CLAM over ABMIL. MIPCL had a balanced accuracy 91.00% (+ 2.88), a weighted F1-score of 91.05% (+ 3.07), a weighted AUROC of 0.9377 (+ 3.19), and a weighted AUPRC of 0.9542 (+ 3.11) (Fig. 2).

**Table 1:** Results from evaluations of ABMIL, CLAM and MIPCL.

Model	Balanced accuracy (%)	F1-Score (%)	AUROC (area)	AUPRC (area)
ABMIL	88.11 [86.85, 89.39]	87.97 [86.63, 89.25]	0.9159 [0.8929, 0.9498]	0.9334 [0.9170, 0.9498]
CLAM	88.81 [87.51, 90.00]	88.70 [87.29, 89.91]	0.9051 [0.8725, 0.9378]	0.9290 [0.9069, 0.9511]
MIPCL	**91.00 [89.94, 92.10]**	**91.07 [90.00, 92.16]**	**0.9435 [0.9255, 0.9615]**	**0.9584 [0.9452, 0.9715]**

The results are the averages across 10 test folds with the confidence intervals in brackets. Both CLAM and MIPCL are compared to ABMIL (baseline). MIPCL was statistically significant over ABMIL across all metrics except AUROC (*p*-values of 0.007, 0.002, 0.053, 0.018); MIPCL was statistically significant over CLAM across all metrics (*p*-values of 0.024, 0.013, 0.002, 0.002). CLAM was not statistically significant over ABMIL on any of the metrics (*p*-values of 0.13, 0.188, 0.784, 0.688). The best results per metric are in bold.

Additionally, our MIPCL model had a lower number of trainable model parameters (298K) than that of both ABMIL (330K) and CLAM (340K). Having that said, all three models were computational efficient with regards to both training and inference. The inference time—from preprocessing to extract features with a pretrained model and to prediction from MIPCL—takes approximately 40 seconds on average per one square cm. The step that accounts for the majority of the computation time is the extraction of features from the pretrained neural network, which also depends on the number of tiles extracted from the original WSI by the preprocessing step.

To further investigate the performance of our model, we looked at the test results from each fold and focused on the cases in which the model scored a high probability of the slide being positive for carcinoma, but the labels were negative for carcinoma. From this analysis, across the stratified 10-fold test sets (for a total of 1805 cases), we found 14 cases for which the case was given an incorrect label (negative for carcinoma) during the data curation process, and the model was in fact correct (positive for carcinoma).

**Figure 3:**
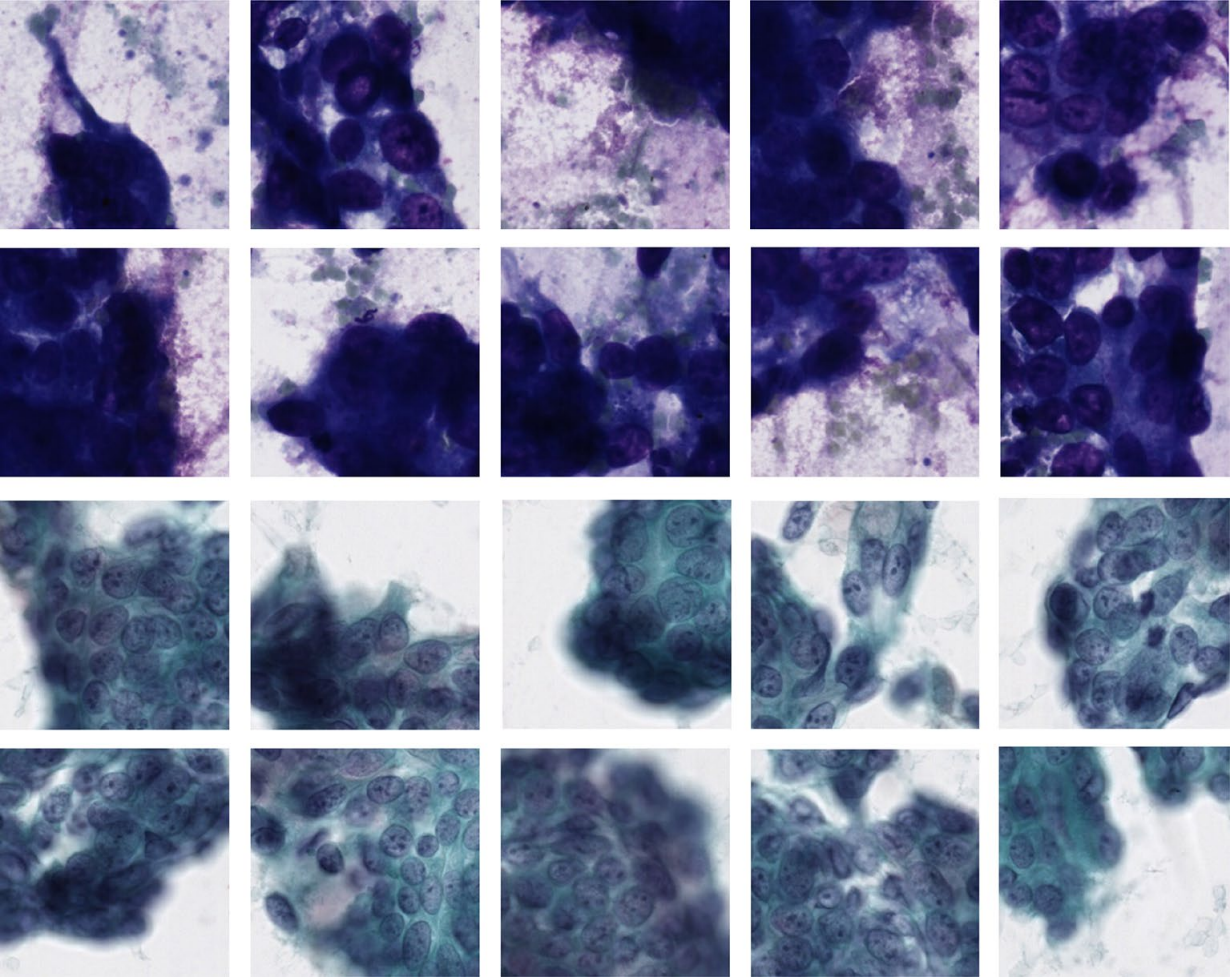
Visualization examples from two cases. The top ten tiles predicted by MIPCL with the highest probabilities for pancreatic ductal adenocarcinoma are shown here. The top two rows are from a Diff-Quick smear, and the bottom two rows are from a Pap smear. The end-user cytopathologist can utilize these visualization tiles to focus on the most high-yield regions on the slide, and simultaneously glean insight with regards to how the model predicted its final decision.

### Visualizing the top tiles/instances for classification

For visualization, we retrieve the tiles for which the model marked as being above a set threshold for the Grad-CAM derived probabilities (Fig. 3) from our proposed framework (Fig. 1). We find this approach to be advantageous for several reasons. Due to the fact that cytopathology consists of cells sporadically interspersed throughout the slide, cytopathologists do not review slides in a continuous fashion, and instead discontinuously jump from collections of cells to collections of cells present on the slides. Moreover, experienced cytotechnologists, when present, triage the slide first and dot with an inking pen areas that are high yield for the cytopathologists to focus on. This drastically improves on efficiency for the cytopathologist and reduces the mental burden.

In like manner, in recovering the most salient tiles (e.g. above a Grad-CAM derived probability) toward the classification from the model for our visualization procedure, we not only get an insight into how the model came to its conclusions, but we are also redirected to areas within the slide that are most salient for classification. This serves a similar function to what the cytotechnologists perform when they triage and dot the areas for the cytopathologist to review. In other words, we obtain model explainability and a helpful screening tool simultaneously.

**Figure 4:**
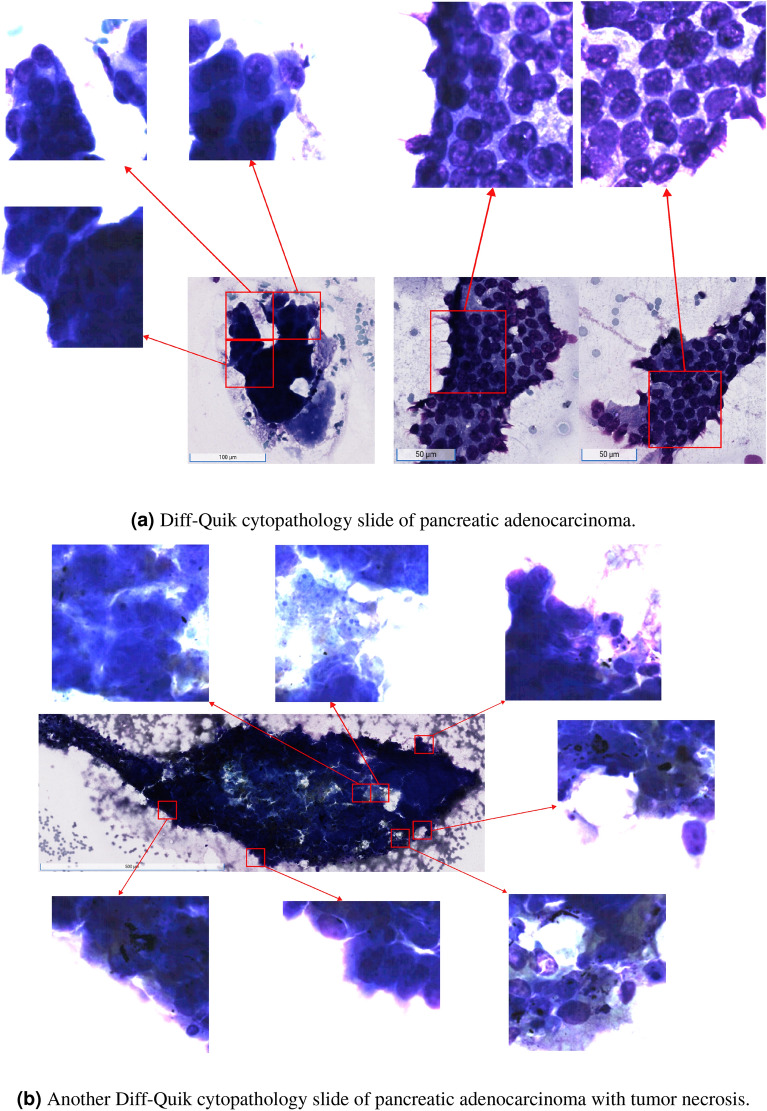
The model learns to attend to regions that converge with what expert cytopathologists look for in making a diagnosis of pancreatic adenocarcinoma. (**a**) In the left column of (A), the model focuses on the areas of this collection of neoplastic cells where the nuclear features are best seen, which is at the periphery. On the right column, the model learned to weight highly areas of disordered architecture, for which, cytopathologists would call a “drunken honeycomb” appearance. (**b**) The model learns to focus on the periphery of tumor necrosis where there are still viable cells. The model doesn’t completely avoid the necrosis, however, and does weight highly areas for which the ghost cells of the tumor necrosis can still be seen (top left tile). The contrast for the tiles have been increased to better see the nuclear features; the model did not have access to these alterations to image characteristics.

Upon visualization the top tiles, we observed that the model was converging on latent features that semantically correspond to the same cytomorphological features that expert cytopathologists also probe for when making a diagnosis of pancreatic adenocarcinoma. The model learned to pick up on variation in nuclear sizes, presence of nucleoli, disorganized cellular architecture, and even learned to focus at the peripheries of a collection of cells (Fig. 4a), where nuclear morphological features of carcinoma are best seen. For example, the model was able to attend to the periphery of an area of tumor necrosis, in which the viable cells are best seen, and still pick up on necrotic ghost tumor cells for which nuclear features can still be made out (Fig. 4b).

We also analyzed the false positive cases from the model. Upon reviewing the tiles/instances with the highest Softmax probabilities from these cases, we observed that the model was localizing in the vicinity of the feature subspace for that of adenocarcinoma, such as large nuclear features, nucleoli, etc. Furthermore, it was picking up features that also happen to be well known pitfalls for cytopathologists, such as areas of gastric contamination, heavy Diff-Quik staining, and acinar cells with strong Diff-Quik staining.

## Discussion

Unlike its surgical pathology counterpart, cytopathology doesn’t have a neat and straightforward distinction between cells/tissue and background. Instead, there are collections of different cell types that are distributed sporadically across the slide. To make matters more difficult, cytopathology utilizes two different staining techniques—Papanicolaou and Diff-Quik smears—which generate differing amounts of artifacts. The slide preparation is also susceptible to drying artifact, which alters the cytology and further compounds the difficulty.

Another major difference between the two is the number of WSIs digitized for a single case. Cytopathology utilizes a Z-plane for routine practice unlike surgical pathology. Therefore, while a surgical pathology WSI consists of only a 2D image, a cytopathology WSI consists of multiple, large 2D images aligned with respect to the Z-axis. With that said, the jury is still out with regards to the number of Z-planes and at what Z-intervals are optimal, or even required, for image analyses. Some studies have reported that, as long as there are enough epithelial cells on the slide, a single Z-plane suffices a la surgical pathology^18–20^. For our study, we found that the a single plane—the initial plane of focus—was efficient and sufficient for the prediction task.

In our study, we reproduced two deep learning models from digital surgical pathology, ABMIL and CLAM, which serve as standards of comparison. Across a variety of performance metrics, the performance between CLAM and ABMIL was equivocal. This overall improvement is not surprising considering that CLAM can be seen as a regularized and data-efficient version of ABMIL. We suspect that CLAM will particularly shine over ABMIL in regimes of low data, just as it was reported for its original surgical pathology classification tasks.

**Figure 5:**
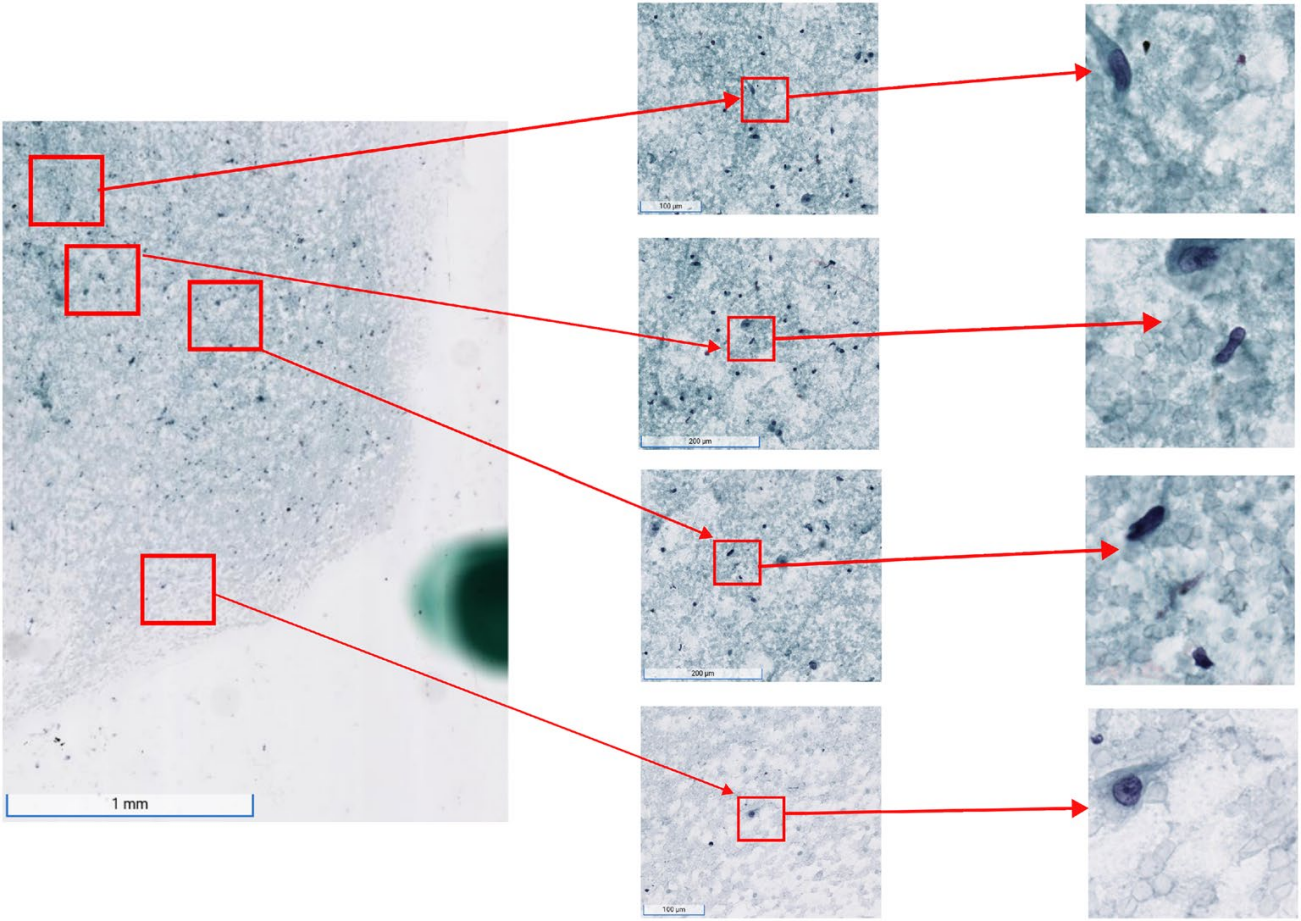
The model is able to pick up on subtle cytomorphological features on small volumetric cells. These can be missed by human experts, particularly when they are fatigued, and the consequences can be costly (missing diagnosis of cancer). The suspicious cells found within the bottom two tiles, for example, account for roughly 0.032% (69.85 $\times$ 31.8 $\upmu ^2$m) of the percent area taken up on a 3.5 $\times$ 2.0 $\hbox {mm}^2$ WSI. The left column is the original recapitulated/regional WSI; the middle column represent zoomed areas of interest that we provide for depiction; the right column showcases the tessellated tiles that were the most salient for a diagnosis of pancreatic adenocarcinoma.

Our model significantly outperformed both ABMIL and CLAM across all metrics. Furthermore, MIPCL identified 14/1805 test cases for which the label was incorrect—model predicts the presence of carcinoma, but the label was negative for carcinoma. In 3/14 of these cases, MIPCL was able to flag tiny volumes of cells that were highly suspicious for malignancy (Fig. 5). Small volumes of cells can easily be passed over, particularly when the expert cytopathologist is fatigued. Therefore, a screening artificial intelligence tool, such as ours, may mitigate cytopathologist fatigue and help minimize false negatives.

Like both ABMIL and CLAM, our framework also proffers model explainability by way of visualization. While inspecting the top tiles/instances that the model attributed towards classification is not new to WSI image analysis, we find that it is especially apropos to cytopathology versus surgical pathology. Cytopathologists and cytotechnologists also scan the slide, looking for the most salient regions that are contributory to the diagnosis. This corresponds to a deep learning model scanning the tiles and selecting out the ones that are most significant towards the final prediction. With regards to model deployment in the real-world, our MIPCL model has fewer number of network parameters than that of both ABMIL and CLAM. This becomes an important factor if these models are put into use in the real world at scale and with time considerations. Altogether, our MIPCL model significantly improves over ABMIL and CLAM across all metrics with model explainability while requiring less computational budget.

In addition to MIPCL, we also proposed a data curation strategy to minimize the resource burden towards collecting and digitizing cytopathology cases, while increasing the number of training examples from the existing slides. While the regional WSIs are not the original full slides, they serve as an effective proxy to the full slide for training and inference. In other words, through our data curation strategy, we augment our dataset from an existing one, maintain the distinctives of high-gigapixel resolution emblematic of digital pathology, and reduce the resource burden of collecting more training examples and digitizing them.

Our study has the potential for immediate real-world applicability with regards to addressing a big need currently in pathology. There has been a continuing growing concern of technical staffing shortage across pathology laboratories. This is particularly acute in cytopathology with severe shortages of cytotechnologists who prepare and triage the cytopathology cases. This results in a reduction of efficiency, leading to a delayed diagnosis for the patient and increasing the burden to the cytopathologist. To this end, our model can effectively reproduce a cytotechnologst slide triage since MIPCL can operate as a performative and fast AI assistant that can simultaneously predict the presence or absence of suspicious cells on the WSI, and highlight subregions on the WSI consising of said suspicious cells. Furthermore, we believe our model has the potential to reduce the number of passes of tissue sampling during the biopsy procedure, which is a function of achieving cellular adequacy and confirmation of lesion localization, due to the model’s high sensitivity even in paucicellular specimen.

### Limitations

The main limitation of this study is that MIPCL did not have an external independent test set (i.e., slides prepared from other laboratories). Smear characteristics of pancreatic FNAs can vary widely, not only across laboratories, but also among the staff who stain the slides. While our dataset did show smear variation, which we would argue is intrinsic, the final gauge of generalizability and robustness to distribution shifts would be on datasets prepared at other institutions/laboratories. The test sets within our study also did not include any original WSI, and acknowledge that we expect our model to perform just as well on them since the main difference between the original and regional WSI is the number of tiles to be processed. However, this was not formally validated in this paper.

Our study also focused on the benign-to-adenocarcinoma axis, and did not evaluate for other disease processes. Therefore, to make our model more complete and robust for real-world deployability, MIPCL would have to be more comprehensive to include other neoplastic and non-neoplastic (i.e., neuroendocrine tumors and chronic pancreatitis), which can be pitfalls when assessing for adenocarcinoma. In other words, distinguishing PDAC from other pathologies would be the next step of progression for MIPCL.

## Methods

### Data curation

The curation process of digital pathology datasets is a resource intensive endeavor. For this study, we devised a data curation strategy that leverages characteristics that are unique to cytopathology for the purposes of faithfully training a deep learning algorithm (i.e., at whole-slide imaging scale) while minimizing the resource burden of the curation process. Within a cytopathology slide, there are distinct geographic regions consisting of normal background cells, cells suspicious for pathology/disease, and/or a combination of the two. Additionally, FNA cases often consist of multiple slide preparations. This is due to the fact that several passes of needle aspiration are attempted to ensure adequate lesional tissue was sampled. These distinct regions of interest across multiple preparations can be largely seen as being independent from one another. We take advantage of these particular features of cytopathology to increase the number of training cases without collecting additional cases.

At our institution, each prepared slide (approximately 4.0 $\times$ 2.0 cm) from a case are first screened by a cytotechnologist who then proceeds to marks regions of interest with a dotting pen. The regions annotated by cytotechnologists include areas considered benign and artifactual/contaminant along with areas concerning for and potentially diagnostic of carcinoma. We randomly sub-sampled these regions (approximately 1.0 $\times$ 1.0 cm) from the whole slides within case, when scanning, to result in data consisting of much higher signal to noise ratio with the best quality and are most representative. We then had pathologists review each sub region after scanning and label according to the data model we used.

For our data curation strategy, we reviewed the dotted regions across all slides from 102 in-house cases, and subsequently filtered these dotted regions into areas positive and negative for adenocarcinoma. Moreover, these regions were selected for variability (preparation artifact, out-of-focus artifact, contamination, and more than one cell type). The patient demographic make up of the 102 cases were the following: 52:45 male:female, ages ranging from 36 to 87 with an average of 69.24, and ethnicities of 74 Caucasians, 14 African Americans, 5 unreported, and 4 Asian Americans. The final diagnosis breakdown of the 102 cases was 76 pancreatic ductal adenocarcinoma, 7 chronic pancreatitis, 8 mucinous cystic neoplasm (these cases didn’t have a final resection diagnosis), 6 intraductal papillary mucinous neoplasm, 1 undifferentiated carcinoma, 1 metastatic renal cell carcinoma, and 1 pancreatic pseudocyst. Due to our institution being a major referral site for pancreatic cancer, the pancreas cases that are seen here skew towards cancer. Our curated dataset reflects this, and the final dataset had approximately a 3:1 ratio of positive to negative for adenocarcinoma.

**Figure 6:**
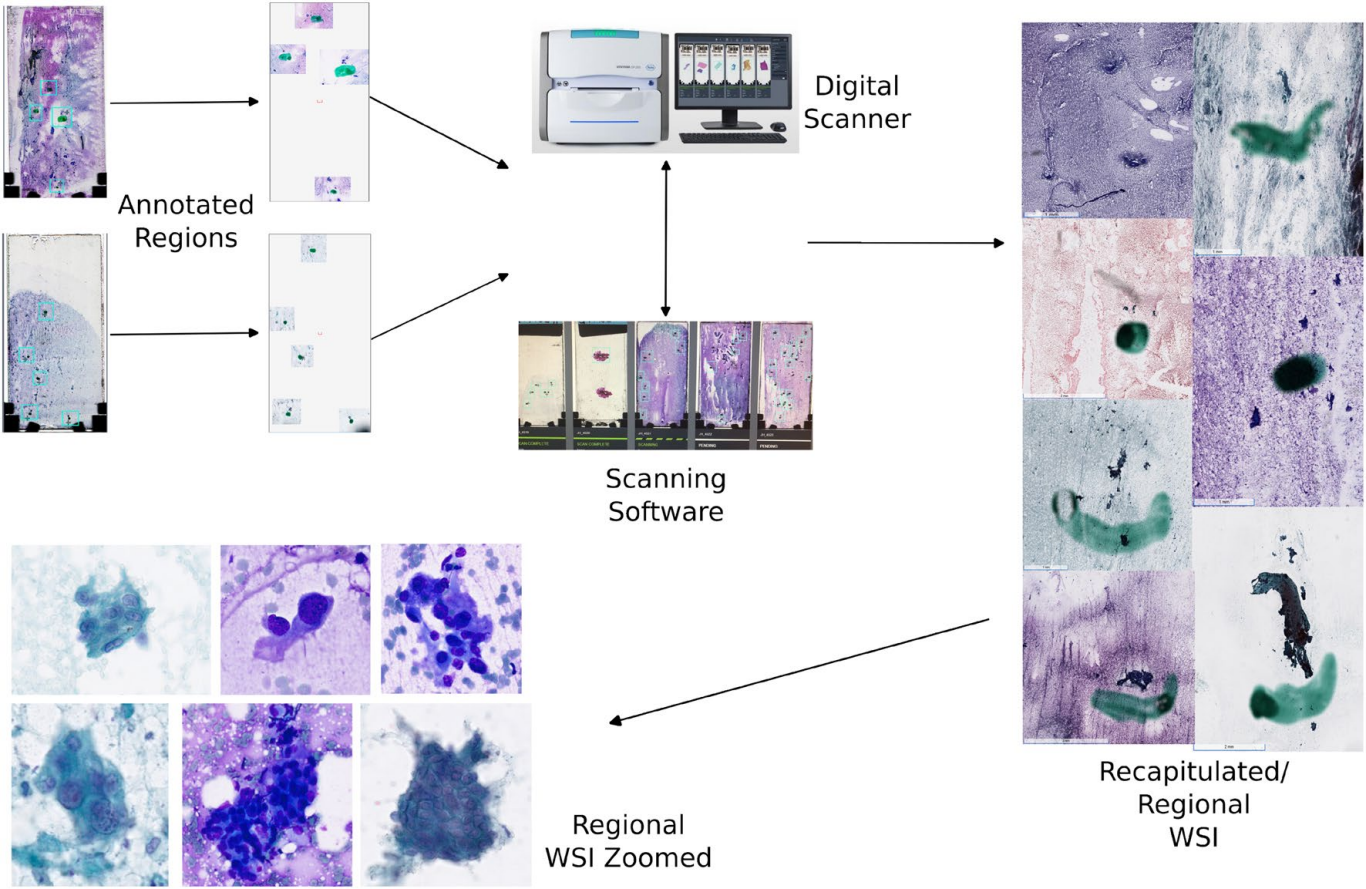
Overview of the data curation processing pipeline. Regional WSI are generated from the original full WSI, indexed by the ink dots from the cytotechnologist who initially screened the case. These regional WSIs faithfully recapitulate WSIs, are sufficient for deep learning training with high-gigapixel resolution, and are a way to increase the training examples per class without collecting more patient samples.

Upon completion of review, these filtered regions were then scanned as regional WSIs with a Hammamatsu digital scanner at 20$\times$ resolution, which were 15,135 by 15,099 (X by Y) pixels on average, and with 13 z-planes (6 above and 6 below the initial plane of focus). Two-thousand gigapixel-scale pancreatic FNA WSIs (Fig. 6) were recapitulated as a result of our curation protocol. The scanned slides, while not the full original WSIs, serve as a faithful proxy for achieving WSI scale. With this strategy, we were able to increase the number of training examples for each class label without requiring the collection of additional patient samples.

### Preprocessing

Cytopathology slides come with abundant noise (Fig. 6) from both the preparation and screening steps. As a result, a straightforward foreground segmentation algorithm is not readily available. During our study, we discovered that a color filter strategy worked best to maximally segment cells of interest while minimizing background noise. The color filter strategy was also able to eliminate the ink dots to prevent biasing the downstream deep learning algorithm. We empirically narrowed our filters to localize to the nuclei and cytoplasm with respect to both Diff-Quik and Pap stains. Moreover, we found that operating in the hue-saturation-value color space, as opposed to the red-green-blue color space, provided tighter and more precise filters.

**Figure 7:**
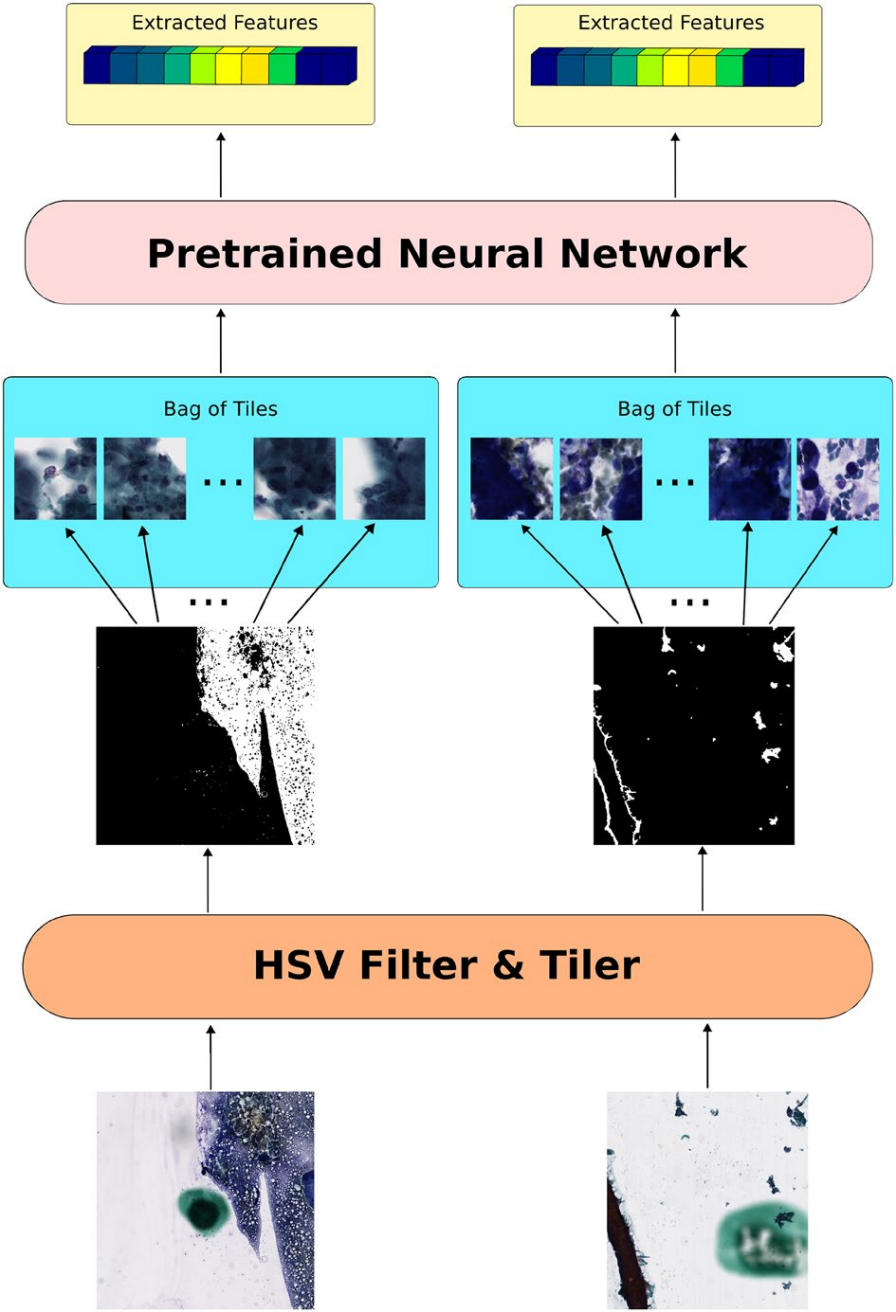
Overiew of the preprocessing workflow. The whole slide images are first masked by a HSV filter that selects for nuclear and cytoplasmic ranges per stain preparation (i.e., Pa vs DiffQuik). The whole slide images are then tessellated with respect to the generated mask into 256 $\times$ 256 pixel tiles.

The original WSI is first masked with the color filter above, and subsequently tessellated into tiles of 256 $\times$ 256 pixels at the derived masked locations (Fig. 7). We then utilized a pretrained neural network (ConvNext^21^) to extract features from these tiles followed by global average pooling to further reduce the dimensionality of the extracted features. The featurized tiles were then stored with h5py^22^ for downstream neural network training.

### Quick primer on multiple instance learning

Applying deep learning for digital pathology images is challenging if only due to the ultra-high resolution. While most studies within computer vision operate on the order of $10^2 \times 10^2$ of pixels, digital pathology images easily range from $10^4 \times 10^4$ to $10^5 \times 10^5$ of pixels. For example, a typical image from the popular computer vision database, ImageNet^23^, averages approximately 400 $\times$ 400 pixels, whereas the scanned images in our dataset average approximately 15,000 $\times$ 15,000 pixels.

The standard operating procedure in deep learning which involve the classification of ultra-high resolution images (e.g. satellite images in remote sensing as another example) use the multiple instance learning (MIL) paradigm^24^. In MIL, instead of receiving a set of instances that are individually labeled, the learning model receives a set of labeled bags (i.e., WSIs), in which each bag contains many instances (i.e., tessellated tiles of a WSI). The standard MIL assumption is that all negative bags contain only negative instances and that positive bags contain at least one positive instance.

One way to think about MIL is that it is a method to manage the statistical independence among the instances within a bag. On the one hand, we want to remain invariant to feature instances that are not relevant to the classification task at hand. On the other, we want to maximize the feature instances that are salient (e.g. positive for carcinoma). In other words, we want the final classifier to correctly predict that the WSI is positive for cancer for which there is only a handful of pixels of cancer regardless of how many healthy cells are also present within the WSI.

In our study, a bag is defined as a set feature vectors generated from the preprocessing pipeline. These features were further embedded unto a lower-dimensional feature space with a simple encoder that consisted of a fully-connected layer, a group normalization layer^25^, a dropout layer^26^, and a Mish^27^ activation layer. Let ${\textbf {{X}}}$ be a bag defined as a set of feature vectors ${{{\textbf {X}}}} = {{{{\textbf {x}}}}_1, {{{\textbf {x}}}}_2, \ldots , {{{\textbf {x}}}}_N}$ where *N* is the number of features. Each instance (i.e. feature vector), ${{{\textbf {x}}}}_i$, in feature space, *F*, can be mapped to a class by a neural network $f: F \rightarrow \{0, 1\}$, where the negative and positive classes (negative and positive for pancreatic adenocarcinoma) correspond 0 and 1, respectively. Therefore, a bag classifier $g({{\textbf {{X}}}})$ is defined as:

1 $$\begin{aligned} g({{\textbf {{X}}}}) = {\left\{ \begin{array}{ll} 1, &{} \exists {{{{\textbf {x}}}}} \in {{{\textbf {X}}}}: f({{{\textbf {x}}}}) = 1; \\ 0, &{} otherwise \end{array}\right. } \end{aligned}$$

### Multiple instance probability contrastive learning (MIPCL)

Due to the presence of gratuitous artifacts and noise in cytopathology specimens, we incorporate an insight from^28^ and introduce a disentangler module into our own network. The disentangler module consists of a fully-connected layer, a group normalization layer, and finally a sigmoid activation head, $\varphi (\cdot )$.

A single class-agnostic activation map per instance is predicted from the disentangler. This effectively produces class-agnostic foreground and background feature representations for each instance. These foreground and background feature instances are then contrastively clustered (Fig. 1) via the InfoNCE loss function (${\mathcal {L}}_{Instances}$)^29^. The *i*-th foreground and background feature representations are defined as:

2 $$\begin{aligned} {{{\textbf {v}}}}^{\,f}_{i} = {{{\textbf {P}}}}_i \otimes {{{\textbf {Z}}}}_i, \quad \hbox {and} \quad {{{\textbf {v}}}}^b_i = (\hbox {1} - {{{\textbf {P}}}}_i) \otimes {{{\textbf {Z}}}}_i, \quad \hbox {respectively.} \end{aligned}$$

Here, ${{{\textbf {P}}}}_i \in {\mathbb {R}}^{NX1}$ is the activated foreground regions (output of $\varphi$ above), $(\hbox {1}-{{\textbf {{P}}}}_i) \in {\mathbb {R}}^{NX1}$ is the background activation map, and ${{{\textbf {Z}}}}_i \in {\mathbb {R}}^{NXC}$ are the embedded features outputted from the simple encoder. *N* indicates the number of instances within the bag, *i* the instance index, *C* the feature dimensionality, and $\otimes$ is Einstein summation. In tuning the temperature hyperparameter for the InfoNCE loss function, we discovered that a value of 0.07 was optimal.

The conceptual idea behind the disentangler module is to have it serve analogously to a highlighter function. In other words, the predicted single class-agnostic activation map resulted from the disentangler module accentuates the cells of interest further to the foreground, and drowns out the remainder within the instance to the background. We then perform Einstein summation between the original instances and the activated foreground. These now activated instances are then put through a gradient-based class activation maps^30^ (Grad-CAM) module. Grad-CAM originally served as a spatial visualization tool to reveal the locations in an image that correspond to the predicted classification by a deep learning model. Grad-CAM is defined as:

3 $$\begin{aligned} L^c_{{\hbox {Grad-CAM}}} = \hbox {ReLU}\underbrace{\Big (\sum _k\alpha ^c_kA^k\Big )}_{\text {linear combination}}, \qquad \alpha ^c_k = \overbrace{\frac{1}{Z}\sum _i\sum _j}^{{\hbox {global average pooling}}}\underbrace{\Bigg (\frac{\delta y^c}{\delta A^k_{ij}}\Bigg )}_{\text {gradients via backprop}} \end{aligned}$$

where $A^k$ is the total feature activation map, $\alpha ^c_k$ is the pooled gradients for class *c* at specified locations, and ReLU is the activating function.

Interestingly, however, Grad-CAM conceptually shares an identical perspective with that of MIL. In identifying locations within the image that correspond to the class label under review, like MIL, Grad-CAM seeks to also manage statistical independence at every pixel location, and outputs essentially a heatmap where the heat corresponds to a particular class. We can then take advantage of this fact to derive the probabilities of the classes for each instance by computing the Softmax from $L^c_{{\hbox {Grad-CAM}}}$.

Once the probability maps are generated, the indices of the instances that have Softmax probabilities above a set threshold for each class, $\delta _0$ (negative for carcinoma) and $\delta _1$ (positive for carcinoma), are retrieved (Fig. 1). We experimented with thresholds of [0.55, 0.65, 0.75, 0.85, 0.95] and found a probability threshold of 0.85 for both $\delta _0$ and $\delta _1$ led to the best results. The model then uses these recovered indices to select the foreground instances, $v_{i}^{\,f}$ (Eq. 2). Finally, the selected instances are pooled with the derived Softmax probabilities and taken to classification (${\mathcal {L}}_{Bag}$) with cross-entropy as the loss function. The two losses are finally summed for a total loss (${\mathcal {L}}_{Total} = (1-\beta )\cdot {\mathcal {L}}_{instances} + \beta \cdot {\mathcal {L}}_{Bag}$). While we did experiment with different weights for the two losses, we discovered that a non-weighted total sum produced the best outcomes.

Therefore, to recap, our MIPCL model (Algorithm 1): (1) first disentangles class-agnostic foreground and background activations for each instance; (2) contrastively clusters these foreground and background activations; (3) Einstein sum the original instances with the foreground activations; (4) selects a subset of the activated instances informed by the Softmax probabilities derived from Grad-CAM; and finally (5) pools these selected instances for final bag-level classification. The optimization algorithm chosen was Adam^31^ with its standard hyperparameters ($\beta _1$ 0.9, $\beta _2$ 0.95, $3x10^{-4}$ learning rate, and $1x10^{-4}$ weight decay). We also utilized OneCycleLR^32^ for the learning rate scheduling policy. For the scheduler, we chose a cosine annealing strategy with cycle momentum. The Softmax probability of the CAM logits of each instance, along with the instance’s tile coordinates, were saved for further post-processing review.

### Reproducing ABMIL and CLAM for cytopathology

We compared our proposed MIPCL to Ilse et al.’s ABMIL^17^ and Lu et. al.’s CLAM^6^. Both ABMIL and CLAM have successfully set the standards of comparison within the domain of cancer classification in digital surgical pathology. Both models were trained and tested on various datasets. ABMIL evaluated on a surgical pathology lymph node dataset for the purposes of detecting breast cancer metastases (CAMELYON16), and reported a F1-Score of 0.901 +/– 0.011 and an AUROC of 0.968 +/– 0.009. CLAM trained and tested on The Cancer Genome Atlas surgical pathology study subsets of renal cell carcinoma and non-small cell lung cancer for cancer subtype classification. CLAM reported AUROCs of 0.991 +/– 0.004 and 0.956 +/– 0.020 for subtype classifcation within renal cell carcinoma and non-small cell lung cancer, respectively. Neither studies, however, looked at cytopathology. For our study, we reproduce both ABMIL and CLAM for our pancreatic FNA dataset.

ABMIL proposes an attention-based pooling for the featurized instances. This attention-based pooling takes the weighted average of instances, in which the weights are determined by a neural network. Additionally, they normalize the instance weights to a sum of 1 to maintain invariance to the size of the bag. Let $H = \{{h_1, \ldots , h_K}\}$ be a bag of K embeddings, then the attention-based pooling is the following:

4 $$\begin{aligned} {{\textbf {z}}} = \sum _{k=1}^K a_k h_k, \qquad a_k = \frac{\exp {\{w^\top \tanh {(Vh_k^\top )}\}}}{\sum _{j=1}^K \exp {\{w^\top }\tanh {(Vh_j^\top )} \}} \end{aligned}$$

where $w\in {\mathbb {R}}^{LX1}$ and $V \in {\mathbb {R}}^{LXM}$ are parameters, and $\tanh$ is a non-linear activation unit for the purposes of including both negative and positive values for proper gradient flow. Cross-entropy is used as the loss function during training.

CLAM is essentially an extension of ABMIL. In addition to utilizing the attention-based pooling (Eq. 3), CLAM also incorporates an instance-level clustering that essentially regularizes the model particularly for lower data volume regimes. Since there are no training labels for each of the instances, the authors use the outputs of a gated attention submodule within the network to generate pseudo-labels for each class. The outputted attention values are then sorted in descending order, and the *B* instances with the highest in-class attention scores receive a positive cluster label, and likewise, *B* instances with the lowest in-class attention scores receive a negative cluster label. The authors use a smooth top-1 support vector machine (SVM) loss, which is based off a multiclass SVM loss, for training. The two losses—from both the attention pooling and instance clustering—are first weighted by a hyperparameter and summed for a total loss.

In our reproduction of both ABMIL and CLAM for our cytopathology classification, we optimized both models with the same optimization algorithm, learning rate scheduler, and weighted dataloader as described for our MIPCL framework above. The same hyperparameters for both the optimizer and scheduler were selected for as well. Furthermore, in our reproduction of CLAM, we found the hyperparameters reported in the original paper ($\hbox {top}_K$ of 8 and a bag weight of 0.7) also worked best for our pancreatic FNA classification.

### Software, hardware, and implementation details

The software libraries used for WSI processing and image preprocessing were pyvips^33^ and OpenCV^34^, respectively. For all things deep learning, we used both PyTorch^35^ and timm^36^ libraries. In particular, the timm library was used for the pretrained neural network (ConvNeXt) that we utilized during our preprocessing. For tile visualization, we used the Matplotlib library^37^. For relevant hardware, we used 4X NVIDIA Tesla V100s for feature extraction with the pretrained ConvNeXt within the preprocessing step, and a single NVIDIA RTX A5000 GPU for training and testing the models.

## Data Availability

The digital pancreatic FNA images used for this study will be made upon request to the corresponding author (baras@jhmi.edu). The dataset will be hosted at https://https://digital.pathology.johnshopkins.edu. Upon account registration and request approval, we will provide a link and access to the dataset.

## Code availability

Our experiments (preprocessing, training, testing, and visualization) can be reproduced through the details and configurations outlined under our “Methods” section. Our implementation for all three models can be found at the following github repository: https://github.com/ansohn88/MIPCL.
